# Supplementary material for: Place-provider-matrix of bystander cardiopulmonary resuscitation and outcomes of out-of-hospital cardiac arrest: A nationwide observational cross-sectional analysis
Source: PLoS One. 2020 May 15;15(5):e0232999. doi: 10.1371/journal.pone.0232999 (PMC7228068; doi:10.1371/journal.pone.0232999)
Supplement: Attachment — Submitted filename: review_PONE-D-19-14833_UrayT.pdf [file pone.0232999.s001.pdf]

Thank you for your work and the opportunity to review the submitted manuscript PONE-D-19-14833, entitled "*Place-Provider-Matrix of Bystander and Outcomes of Out-of-hospital Cardiac Arrest: A Nationwide Observational Cross-Sectional Analysis*" by Kim D. et al.

It addresses the pressing topic of bystander-CPR and location of CA, and tries to depict room for improvement. Results are drawn form over 70,000 included patients.

major concerns:

Overall, the manuscript would profit from a thorough grammatical revision by an English native speaker.

Statistics:

The authors state in the results and the discussion section, that CPR at a public place with trained responders (TR) shows the highest OR of survival. In contrast, in Table 1 and table 3, survival to discharge after CPR of TR in public with good CPC is 11.3%, whereas survival after layperson bystander CPR in public is 15%. - Please clarify, report these results in table 1 by comparing different percentage including statistical differences (*p*). Also, the whole discussion has to be adapted.

Is theterm "trained responders/TR" a new definition created by the authors? Please clarify

Line 78- It is not clear, if TRs are called to the scene as first responders or are already on the scene by chance and perform as bystanders. Please clarify.

Per Utstein definition [1], first responders like firefighters or police officers are not "bystanders", since they are part of an organized emergency response system. Therefore, the used definition of "3 types of bystanders" (line 79) should be revised if part of the first responder system.

Reference:

1. *Cardiac Arrest and Cardiopulmonary Resuscitation Outcome Reports: Update of the Utstein Resuscitation Registry Templates for Out-of-Hospital Cardiac Arrest: A Statement for Healthcare Professionals From a Task Force of the International Liaison Committee on Resuscitation. Perkins GD et al. Resuscitation. 2015 Nov;96:328-40*

Minor concerns:

Abstract:

- Line 55. Please add an explanation for the abbreviation “FR” used in line 68.

Please rephrase the conclusion of the abstract.

Introduction:

- Lines 76-77 are unclear / should be revised by a native speaker.

- Lines 86-88 and 91-92 should be re-written (unclear).

- Is the PPM a definition / concept that the authors have newly created or does it already exist per definition? This should be clarified.

Methods:

- The authors should re-consider depicting the study as "prospective" - was data in fact collected prospectively for the study purpose between 2012 and 2017? In lines 135-136 it is also stated that data derive from a (retrospectively assessed) registry.

- Lines 112-119: This paragraph should be shortened.

- Does the study have a positive vote by an Ethics Committe or only the approval of the "review board" of the study hospital?

- What exactly was the pool for patient inclusion - was it access to a nationwide database as described in lines 135-136? This is unclear since expressions such as "the study hospital" are repeatedly used throughout the manuscript (this would suggest only one study centre) and since it seems the study only has "approval of the review board of the study hospital" (would it not be necessary to obtain a nationwide approval by multiple Ehtics Committees according to GCP?).

- Why were only patients over the age of 19 years included in the final analysis? If this is due to local requirements, please specify.

- Line 171. Please add an appropriate reference to “Utstein factors” (see above).

Discussion:

- Line 232 [... previous research outcomes in other studies]: Please cite and give examples.

- Given your discussion about poorer outcomes in the group with CA occurring at home, more literature comparison should be implemented and therefore strengthen your arguments (e.g. Jorgenson et al. on the use of privately owned AEDs: 10.1016/j.resuscitation.2012.09.033).

- Line 240 and following paragraphs: You mention dispatcher-assisted BLS - please comment on the current situation in your country - does it already happen or do you just propose it?

- In fact, it is not true that previous studies could not show benefits of e.g. police-first-responder systems as stated in line 256. The cited study by Husain et al. explicitly states that in their collective, "survival from out-of-hospital cardiac arrests increased with the implementation of police AED programs".

- Line 247: the authors state that the home TR group shows equivalent outcomes with the Public-TR group. In table

- Line 250/251: sentence not clear, please rephrase.

- Line 265: Please add more up-to-date references about the topic of circadian variation of OHCA.

- Concerning the day/night findings, the statement in lines 270-271 [A significantly...] is unclear and needs to be rewritten.

- Please state the - in your opinion - novelty of your findings.

Limitations:

- Are patients also followed-up clinically until hospital discharge as part of your registry? Or did you only - as stated - deduct CPC-levels from general documentation such as discharge papers? This is susceptible to considerable bias and should be emphasized more, also in the "Methods" section.

- Why would "CPR protocols and medications be very different from North America or Europe" (lines 291-292) when you stated before you adhere to current international CPR guidelines?
