# Supplementary material for: Place-provider-matrix of bystander cardiopulmonary resuscitation and outcomes of out-of-hospital cardiac arrest: A nationwide observational cross-sectional analysis
Source: PLoS One. 2020 May 15;15(5):e0232999. doi: 10.1371/journal.pone.0232999 (PMC7228068; doi:10.1371/journal.pone.0232999)
Supplement: Attachment — Submitted filename: Authors reply to reviewers comments _ V2.docx [file pone.0232999.s003.docx]

**Author’s reply to reviewers' comments:**

On behalf of authors, thank you for the very valuable comments by the reviewers on our paper. I am very sorry for late revision. We have been fighting with the COVID-19 pandemic for the last two months.

.

We have attempted to address every point commented on by reviewers in the revised manuscript. While we believe that we have addressed all of the reviewers’ concerns, we would be more than pleased to write additional revisions if needed.

We highlighted all changes in red. Author’s answers or explanations are in blue.

Correspondent author

Sang Do Shin, MD, PhD

Ms. Ref. No.: PONE-D-19-14833

Title: Place-Provider-Matrix of Bystander and Outcomes of Out-of-hospital Cardiac Arrest: A Nationwide Observational Cross-Sectional Analysis 

Reviewers' comments: 

Major concerns:
- The manuscript (including the abstract and tables/figures) still is in definitive need of a thorough grammatical revision by an English native speaker. It is not fit to be read by an international audience at the moment.

(ANSWER) Thank you for the review and comments. The manuscript was revised again for grammatical revision by native speakers.

- The matter of the term “trained responder” is still not resolved. According to Utstein guidelines that should be adhered to, first responders are not bystanders. I understand that in your communities, there are trained persons that work in environments that are likely to be place of a CA. If a CA happens there, then I would call the CPR providers “bystanders”, even if they are specially trained. BUT if they are called or dispatched in any way, they are called “first responders”.

(ANSWER) Thank you for the review and comments. We tried to explain the term “trained responder” more specifically in the introduction and method paragraph. We added brief explanation of trained responder in Line 84-88, 118-120 and 124-128.

(Revision: Introduction, line 84-88)

Trained responders (TRs) refer to a specific group of individuals who have a high chance of encountering cardiac arrest in daily life because of occupational characteristics and training for CPR situations but who are not part of the officially organized emergency response system in a community. In general, TRs fall between a bystander and first responder according to the Utstein definition of provider.

(Revision: Method, line 118-120)

According to the 2004 National Emergency Medical Services (EMS) Act, school teachers, sports instructors, public transportation vehicle drivers, safety guards of national parks, and policemen are required to receive CPR education to encourage bystander CPR.

(Revision: Method, line 124-128)

TRs are not first responders who respond to medical emergencies in an official capacity as part of an organized medical response team. Rather, they are similar to bystanders but have been trained in CPR because of the higher chance of encountering CPR situations due to occupational characteristics. Therefore, TRs do not have the duty of official calls or dispatches from EMS systems. They voluntarily participate in the CPR situation.

- I think what you should focus on is: 1) Introducing a detailed definition and re-writing the paragraphs where this process is described. Also, describe the calling / dispatching process of TRs in detail. At the moment, it is very confusing. 2) Add a sub-group analyses of a) the cases in which the TRs were real “bystanders” and b) the cases where they were called/dispatched to a CA event. By this, a little structure would be added in this rather confusing topic.

(ANSWER) Thank you for the review and comments. The revised paragraphs in the above comment can be a reply for this comment. No different in calling and dispatching process of TRS. Just we can identify the TR groups from the EMS CPR registry. The dispatch for CPR is under the same protocol. You asked a subgroup analysis for the case in which the TRs were “real bystander”. But we have analyzed the bystanders as subgroup such as “Trained responder, Family bystander, and Layperson bystander” The Layperson bystander and Family bystanders are part of general (real) bystanders, not trained bystanders

- I still do not fully understand the course your study took when it comes to Ethics Votes. Was the whole registry approved by a proper Ethics Board that is in accordance with international good scientific practice guidelines or was your study in particular approved? If your “review boards” are Ethics Boards, please state so. If not, you might have to present a proper Ethics’ Vote.

(ANSWER) Thank you for the review and comments. The OHCA database had been constructed by the government par (Korea Centers for Disease Control and Prevention). When a researcher wants to use the database for a research, he or she should get permission of data use using a data request form and Korea CDC review and decide to permit or not the use of data. When a research request the data, he or she also get approval of the IRB at each institution. Our institutional review board has reviewed and approved our specific research.

- It is a clear limitation that neurological outcomes are derived from registry data rather than clinical follow-up, as you explained after my initial question. Please add this to your Limitations section.

(ANSWER) Thank you for the review and comments. We added additional explanation of limitation in Line 327-328.

(Revision: Limitation)

Furthermore, neurological outcome data were derived from a registry rather than clinical follow-up. The limited follow-up could result in potential bias in regard to outcomes.

- I was not aware of the EMS situation in South Korea, thank you for the insight. However, also this must be added to the Limitations section because due to the limited comparability to other countries (e.g. in Europe or the USA), also your results are of limited applicability internationally.
(ANSWER) Thank you for the review and comments. We added additional explanation of limitation in Line 329-333.

(Revision: Limitation)

Fifth, this study was performed in a study setting with a different levels of EMS service. CPR protocols and available medications at the prehospital stage would be different from those in North American and European countries according to national legislation and the local EMS Act. The lack of comparability of the EMS system to other countries would limit international applicability.

Minor concerns:
- My request concerning the inclusion of only patients over 19 years aimed at the – in my opinion – international definition of “adults” for people over 18 years and not 19 – please state why you chose 19.

(ANSWER) Thank you for the review and comments. We changed inclusion criteria age to over 18 years old. The relevant paragraphs of methods, results, and discussion were revised and Tables and Figure were changed due to the criteria.

- The sentence “The OHCAs…” (lines 239-240) is still not clear, please re-phrase.

(ANSWER) Thank you for the review and comments. We re-phrased the sentence in line 248-249.

(Revision: Discussion)

In this study, both outcomes showed higher rates in public places than in home settings, which was independent of bystander type.

- You might want to add the contradicting results of up-to-date literature to your statement that time-of-day influences outcomes (for example Schriefl C et al., Time of out-of-hospital cardiac arrest is not associated with outcome in a metropolitan area: A multicenter cohort study. Resuscitation 2019).

(ANSWER) Thank you for the review and comments. We added additional explanation in line 308-309.

(Revision: Discussion)

Recent research on circadian differences in OHCA reported that there is no relationship between OHCA outcomes and arrest time.
